# Supplementary material for: T-shaped alignments integrating HIV-1 near full-length genome and partial pol sequences can improve phylogenetic inference of transmission clusters
Source: PLoS Comput Biol. 2025 Nov 25;21(11):e1013676. doi: 10.1371/journal.pcbi.1013676 (PMC12685204; doi:10.1371/journal.pcbi.1013676)
Supplement: S1 Table — Each row represents a different mixture of wgs and pol sequences, going from 100% pol to 100% wgs. Each column represents a bootstrap threshold for clustering, with the subcolumns indicating the mean number of clusters and mean cluster size found at that bootstrap. Cluster number was averaged over all samples for a given mixture, and cluster size was averaged over both all cluster sizes in a given sample, and over all samples. (DOCX) [file pcbi.1013676.s001.docx]

Cluster statistics by mixture and threshold.

| Mixture | Bootstrap | | | | | | | | | | | |
| --- | --- | --- | --- | --- | --- | --- | --- | --- | --- | --- | --- | --- |
|  | 70 | | 80 | | 85 | | 90 | | 95 | | 99 | |
|  | Number | Size | Number | Size | Number | Size | Number | Size | Number | Size | Number | Size |
| pol | 255.8 | 3.4 | 256.2 | 3.1 | 251.5 | 2.9 | 239.5 | 2.7 | 209.6 | 2.5 | 149.1 | 2.4 |
| wgs10 | 251.5 | 3.4 | 252.1 | 3.1 | 245.2 | 2.9 | 232.7 | 2.8 | 206.3 | 2.6 | 150.6 | 2.4 |
| wgs20 | 247.4 | 3.4 | 244.5 | 3.0 | 237.1 | 2.9 | 222.3 | 2.7 | 195.7 | 2.5 | 144.3 | 2.4 |
| wgs30 | 245.8 | 3.3 | 240.0 | 3.0 | 232.2 | 2.9 | 218.4 | 2.7 | 190.2 | 2.5 | 140.9 | 2.4 |
| wgs40 | 248.2 | 3.3 | 241.3 | 3.0 | 232.2 | 2.8 | 215.7 | 2.7 | 188.2 | 2.5 | 139.0 | 2.4 |
| wgs50 | 247.3 | 3.2 | 240.2 | 3.0 | 231.1 | 2.8 | 214.7 | 2.7 | 185.9 | 2.5 | 135.7 | 2.4 |
| wgs60 | 247.0 | 3.3 | 239.9 | 3.0 | 231.0 | 2.8 | 215.5 | 2.7 | 187.1 | 2.5 | 139.7 | 2.4 |
| wgs70 | 247.3 | 3.3 | 240.6 | 3.0 | 231.1 | 2.9 | 215.0 | 2.7 | 188.7 | 2.6 | 144.7 | 2.4 |
| wgs80 | 248.4 | 3.4 | 243.3 | 3.1 | 235.4 | 2.9 | 221.4 | 2.8 | 197.0 | 2.6 | 151.9 | 2.5 |
| wgs90 | 249.5 | 3.5 | 247.6 | 3.1 | 241.3 | 3.0 | 229.4 | 2.9 | 206.5 | 2.7 | 164.9 | 2.6 |
| wgs95 | 249.3 | 3.5 | 250.4 | 3.2 | 245.2 | 3.1 | 235.5 | 2.9 | 214.9 | 2.8 | 175.4 | 2.6 |
| wgs99 | 251.6 | 3.6 | 253.0 | 3.3 | 249.6 | 3.1 | 240.9 | 3.0 | 222.2 | 2.8 | 181.8 | 2.6 |
| wgs100 | 253.9 | 3.6 | 255.9 | 3.3 | 253.0 | 3.2 | 243.7 | 3.0 | 226.0 | 2.8 | 185.6 | 2.7 |
| Mean number of clusters and cluster size at different bootstrap thresholds and mixtures. Each value is the mean over the set of mixture samples. | | | | | | | | | | | | |
