# Supplementary material for: T-shaped alignments integrating HIV-1 near full-length genome and partial pol sequences can improve phylogenetic inference of transmission clusters
Source: PLoS Comput Biol. 2025 Nov 25;21(11):e1013676. doi: 10.1371/journal.pcbi.1013676 (PMC12685204; doi:10.1371/journal.pcbi.1013676)
Supplement: S2 Table — Each row represents a different mixture of wgs and pol sequences, going from 100% pol to 100% wgs. Each column represents a bootstrap threshold for clustering, with the subcolumns indicating the mean number of clusters and mean cluster size found at that bootstrap. Cluster number was averaged over all samples for a given mixture, and cluster size was averaged over both all cluster sizes in a given sample, and over all samples. (DOCX) [file pcbi.1013676.s002.docx]

Cluster statistics by mixture and threshold.

| Mixture | Bootstrap | | | | | | | | | | | |
| --- | --- | --- | --- | --- | --- | --- | --- | --- | --- | --- | --- | --- |
|  | 70 | | 80 | | 85 | | 90 | | 95 | | 99 | |
|  | Number | Size | Number | Size | Number | Size | Number | Size | Number | Size | Number | Size |
| pol | 24.4 | 3.3 | 23.8 | 3.0 | 22.6 | 2.9 | 19.5 | 2.7 | 14.5 | 2.4 | 8.0 | 2.2 |
| wgs10 | 24.3 | 3.5 | 24.0 | 3.2 | 22.9 | 2.9 | 20.5 | 2.7 | 15.8 | 2.6 | 10.2 | 2.3 |
| wgs20 | 24.0 | 3.6 | 23.7 | 3.2 | 23.0 | 3.0 | 20.1 | 2.8 | 15.4 | 2.6 | 9.9 | 2.4 |
| wgs30 | 24.1 | 3.6 | 23.6 | 3.2 | 23.0 | 3.1 | 20.1 | 2.9 | 15.2 | 2.7 | 9.8 | 2.4 |
| wgs40 | 23.3 | 3.7 | 22.8 | 3.3 | 21.8 | 3.1 | 19.8 | 3.0 | 16.7 | 2.7 | 10.5 | 2.5 |
| wgs50 | 23.0 | 3.8 | 22.9 | 3.3 | 22.1 | 3.1 | 20.2 | 3.0 | 16.7 | 2.8 | 10.9 | 2.5 |
| wgs60 | 22.0 | 3.9 | 22.3 | 3.4 | 21.7 | 3.2 | 20.9 | 3.0 | 17.3 | 2.8 | 11.6 | 2.6 |
| wgs70 | 23.5 | 3.8 | 22.9 | 3.4 | 21.8 | 3.2 | 20.2 | 3.1 | 16.4 | 2.9 | 12.1 | 2.6 |
| wgs80 | 21.9 | 4.0 | 22.2 | 3.6 | 21.7 | 3.5 | 21.0 | 3.2 | 18.1 | 3.0 | 12.9 | 2.7 |
| wgs90 | 22.4 | 4.1 | 22.7 | 3.7 | 22.9 | 3.4 | 21.3 | 3.3 | 18.6 | 3.2 | 14.4 | 2.9 |
| wgs95 | 23.1 | 4.1 | 23.7 | 3.7 | 23.0 | 3.5 | 22.0 | 3.3 | 20.1 | 3.2 | 15.8 | 2.9 |
| wgs99 | 23.1 | 4.3 | 24.7 | 3.7 | 24.4 | 3.5 | 23.2 | 3.3 | 20.7 | 3.2 | 17.2 | 3.1 |
| wgs100 | 22.3 | 4.4 | 23.1 | 3.9 | 23.3 | 3.6 | 22.1 | 3.4 | 19.9 | 3.3 | 16.6 | 3.2 |
| Mean number of clusters and cluster size at different bootstrap thresholds and mixtures. Each value is the mean over the set of mixture samples. | | | | | | | | | | | | |
