# Supplementary figures and images for: T-shaped alignments integrating HIV-1 near full-length genome and partial pol sequences can improve phylogenetic inference of transmission clusters
Source: PLoS Comput Biol. 2025 Nov 25;21(11):e1013676. doi: 10.1371/journal.pcbi.1013676 (PMC12685204; doi:10.1371/journal.pcbi.1013676)

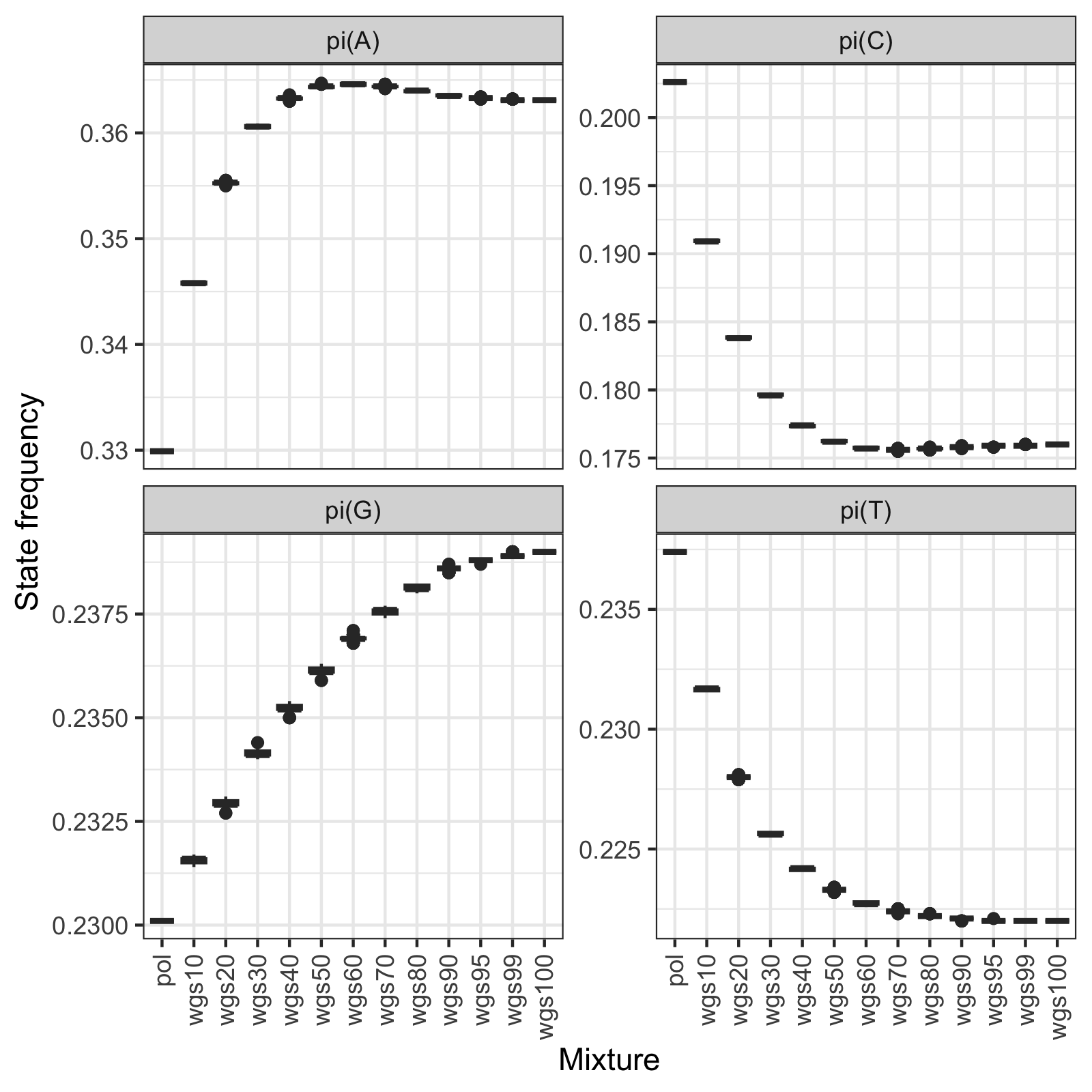

Supplement: S1 Fig — The figure explores the impact of the 4 different base frequency proportions (pi(A), pi(C), pi(G), pi(T), gray bar on top of each panel) in our choice of substitution model for IQTree (GTR+F+I+G4). Proportion is on the Y-axis, and the different mixture datasets are ordered from pol to wgs100 on the X axis. Boxes in panels indicate the central 50% (top and bottom of boxes) and the median (thicker black line in boxes) of the proportions, whiskers indicate the range, and dots indicate outliers. There is a monotonic relationship between relative rate values and wgs proportion beginning with wgs10, when wgs sequences are introduced. (TIFF) [file pcbi.1013676.s003.tif]

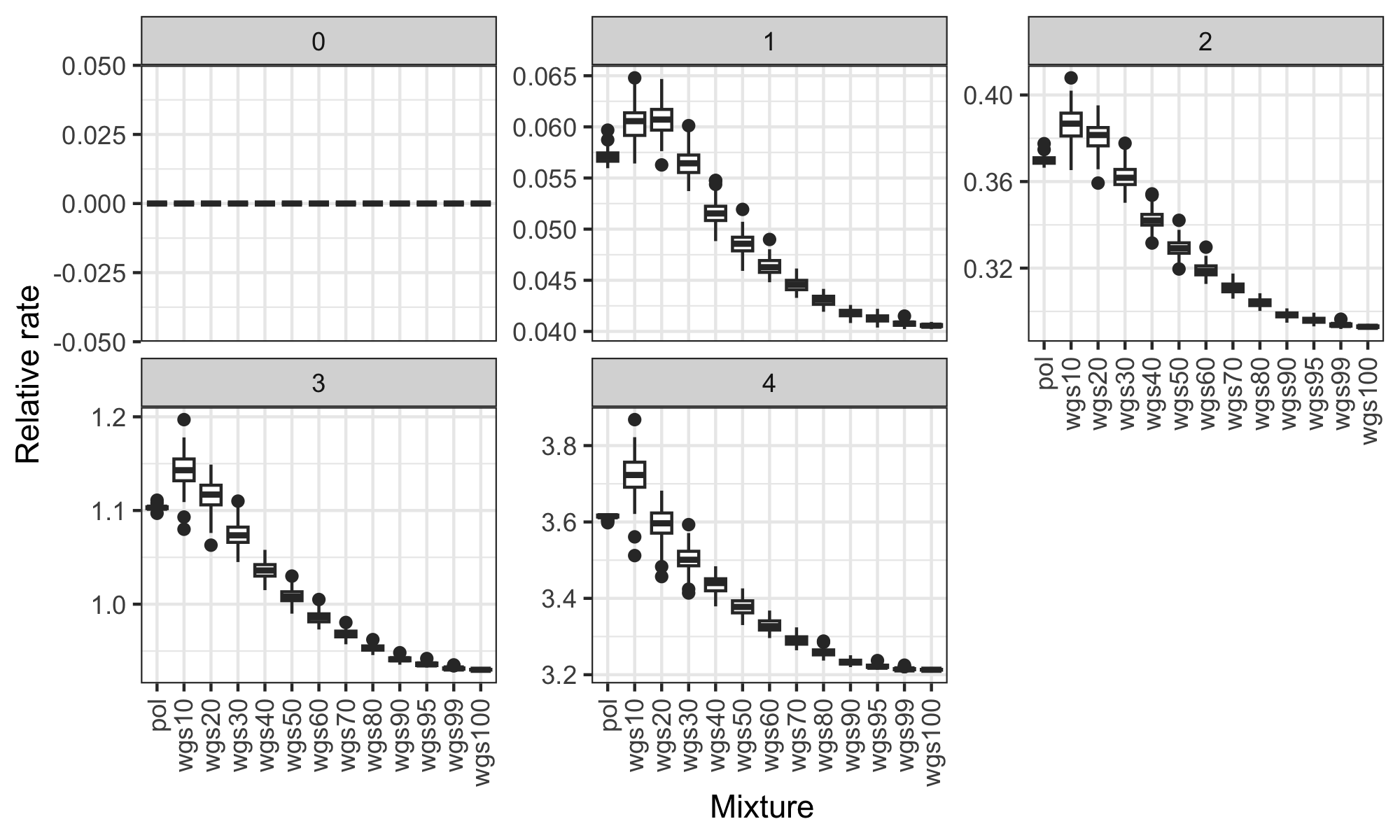

Supplement: S2 Fig — The figure explores the impact of the 4 different gamma rate categories (gray bar on top of each panel) in our choice of substitution model for IQTree (GTR+F+I+G4). Rate is on the Y-axis, and the different mixture datasets are ordered from pol to wgs100 on the X axis. Boxes in panels indicate the central 50% (top and bottom of boxes) and the median (thicker black line in boxes) of the proportions, whiskers indicate the range, and dots indicate outliers. There is a monotonic relationship between relative rate values and wgs proportion beginning with wgs10, when wgs sequences are introduced. (TIFF) [file pcbi.1013676.s004.tif]
