# Supplementary material for: T-shaped alignments integrating HIV-1 near full-length genome and partial pol sequences can improve phylogenetic inference of transmission clusters
Source: PLoS Comput Biol. 2025 Nov 25;21(11):e1013676. doi: 10.1371/journal.pcbi.1013676 (PMC12685204; doi:10.1371/journal.pcbi.1013676)
Supplement: Attachment — Submitted filename: Reviewer_comments.docx [file pcbi.1013676.s005.docx]

Reviewer comments PLOS Computational Biology

Title: T-shaped alignments integrating HIV-1 near full-length genome and partial pol sequences can improve phylodynamic inference of transmission clusters

Manuscript Number: PCOMPBIOL-D-25-00292

Originality:

- The authors successfully synthesized pre-existing tools and approaches in a feasible manner tackle a pre-existing problem. The methods proposed are not new but the approach is simple, conclusive and beneficial.

Innovation:

- The findings show potential for an innovative approach. No innovation regarding methodology.

Significance: Improvable when points are addressed but given.

Importance:

- nFL Sequences are becoming more common and trimming sequences can lead to loss of information and pose technical challenges.
- Patients that have already provided a sequence might not provide another sample due to various reasons or sequencing might fail due to viral suppression.

Methodology: Coherent, conclusive. A few points ameliorable.

Evidence for conclusions: Conclusive but possible confounders to be explored.

I want to thank the editor for the opportunity to review the research article by Guang et al. which proposes an approach to include the available genetic information of nFL genomes into standard pol alignments for HIV-1 molecular epidemiology and investigate experimentally the potential benefit of this approach for inference of HIV-1 transmission clusters. They used publicly available nFL HIV-1 subtype B sequences from the HIV Los Alamos database and created 10 different mixtures of nFL and sequences trimmed to pol only by introducing gaps into the alignment. For cluster detection they used IQtree for phylogenetic inference and a midpoint clustering algorithm with bootstrap cut-off values, while exploring the results for different mixtures of fractions for nFL sequences and cut-off values for bootstrap and compare the adjusted mutual information regarding clusters and tree topology. They found that only after the cut-off of 50% nFL sequences in the alignment, compared to the pol-only alignment, inference of HIV-1 transmission clusters is improved. The manuscript addresses an objective in the research field of molecular epidemiology, however, to enhance the strength of the manuscript I suggest the following revisions.

Major Points to address:

- Methodological:
  - Regarding the entity of the available sequences, the Los Alamos database harbors a variety of methods that have been used to derive those sequences. Unity in sequence acquisition is practically infeasible, however, one major point to address should be that the authors should focus on either sequences derived from viral RNA or proviral DNA and removing the other. Has it been ensured that only viral RNA sequences are used? If not, I recommend doing so, since in cases of mixture, when proviral sequences have been used, an increase in similarity might be spurious due to the increase of similarity of sequences across entity rather than mutual sequence information (e.g. G-A mutations are more common in proviral DNA and might lead to close phylogenetic signal in nFL sequences due to hypermutation instead of genomic evolution). Alternatively, instead of completely removing proviral DNA sequences, the authors could check and include only intact sequences (e.g., using the ProSeqIT tool or HYPERMUT). I acknowledge the fact that this procedure should overall not impact the results substantially, since the analysis is of comparative nature in regards of the initial phylogenetic reconstruction, however, I believe that adapting this would increase the strength of the manuscript.
  - The authors introduced gaps when constructing the T-shaped alignments. Since the presented manuscript provides an experiment approach to the methodology, has it been tried to introduce ambiguous nucleotides (‘N’) instead of gap characters and rerun the analysis to assess differences?
  - When incrementally increasing the proportion of wgs sequences into the T-shaped alignment I did not understand from the method section if only the proportion of the wgs sequences was increased or, starting from the lowest fraction of wgs, partial/pol sequences were ‘filled-up’ incrementally, ensuring that the same wgs are also used in the next step. This would increase comparability and potentially explain the increase in accuracy metric when using 50%.
- Results:
  - L115, Besides the wgs50 tree, there is no supporting material for the claims made in this paragraph, which would be necessary for readers to verify the conclusions from these claims. Referring to the other point, one should also distinguish between proviral and viral sequences and check this again.
  - Figure 6 is missing.
  - Please ensure, also in the supplementary figures, that the x and y axis (and headers) have meaningful labels (e.g. Change ‘Value’ with the metric).
