# Supplementary material for: T-shaped alignments integrating HIV-1 near full-length genome and partial pol sequences can improve phylogenetic inference of transmission clusters
Source: PLoS Comput Biol. 2025 Nov 25;21(11):e1013676. doi: 10.1371/journal.pcbi.1013676 (PMC12685204; doi:10.1371/journal.pcbi.1013676)
Supplement: Attachment — Submitted filename: reviewer_response_v3.docx [file pcbi.1013676.s006.docx]

We thank the reviewers as well as the editor for their very helpful and informative comments and suggestions. Taking all comments into account and making the revisions requests, we both strengthened the manuscript and its conclusions and discovered some new results. We have updated the manuscript accordingly, and below provide our responses to each comment raised by the editor and reviewers, with our replies presented in indented text. We also provide the manuscript with highlighted changes made to the original version, and an unmarked version of the revised paper without tracked changes (all line references in our responses refer to this clean version). Finally, we have made some additional general changes throughout the manuscript to reflect the edits made in response to the editor and reviewer comments. Thank you again for allowing us to revise the manuscript, and we hope it is now suitable for publication in PLOS Computational Biology.

**Editor comments:**

The two reviewers have raised several important points that should be addressed. In addition, I have the following comments:
1) In the "Author Summary", the authors write that a novel approach for analyzing the spread of HIV-1 is explored. This is somewhat misleading, as this manuscript does not look into HIV-1 transmission, but clustering results in a convenience samples. No conclusions about any HIV transmission dynamics can be drawn here. Similar, the lines 226-229 in the Discussion concerning public health go in my opinion far beyond what was done here, a recommendation concerning cluster certainty should be avoided.

We thank the editor for this helpful comment and have adjusted our manuscript accordingly. The first sentence now reads:

“We introduce and explore a novel approach to analyzing HIV-1 clustering through advanced genomic sequence analysis techniques.”

We have also edited line 336-339 (formerly 226-229) to now read: “Since phylogenetic trees enhance pure genetic distance-based methods and can supplement contact tracing in public health settings, using nFL HIV-1 genome sequences may provide additional measures of cluster certainty when performing phylogenetic-based analyses”

We hope this addresses the editor’s concerns.

2) For this manuscript, as this describes mainly the workflow for exploring the impact of different fractions of full-length genomes, having the "Methods" section before the "Results" section would improve the reading flow.

We agree, and have changed the order of section in the manuscript accordingly.

3) Methods: To me it is not clear - also Reviewer 1 pointed this out - what was shuffled 1000 times randomly. This is a crucial point: Did you shuffle the order of sequences every time, and thus different sequences were "cropped" to consist of pol only? I am worried that if this was not shuffled properly, and thus always the same sequences were cropped, this might lead to artificial clustering due to sequences in the Los Alamos Database being more similar. Please clarify this aspect in the discussion. You might also think about a "methods" Figure clearly explaining the algorithm and different measures you applied to assess the impact of the fraction of full-genomes.

We shuffled the order of the sequences every time before cropping. Thank you for the suggestion of a methods figure; we have now included one (Figure 1) in the manuscript. To clarify the methods, we have also made the following changes in the Methods section:

- Deleted the line: “This was repeated 1000 times by shuffling the taxa to be masked randomly each time, with each separate run taken as an individual sample”.
- Rephrased the paragraph describing the mixtures: “For both the full alignment dataset and the subset dataset, we created 100 alignment samples by shuffling the order of the taxa in the alignment randomly with a different seed each time (Fig 1B). For each sample, we created 13 datasets with different mixtures of nFL (referred to as wgs for whole genome sequences) and pol sequences by using the HIV-1 HXB2 (Genbank accession number K03455) genome coordinates to replace (mask) all characters outside of the protease-reverse transcriptase (PRRT) region with gaps according to the mixture of pol specified (Fig 1C). Eleven of the datasets had mixtures in increments of 10% of the taxa being nFL in the alignment starting from 0%, i.e. from 10% up to 100%, while 2 of the datasets were were mixtures of 95% nFL and 5% pol and 99% nFL and 1% pol, respectively. Alignments were labeled by the proportion of nFL sequences in the mixture, i.e. wgs40 represents 40% wgs and 60% pol sequences. This created 13,000 alignment datasets total.”

4) Selection of all available Los Alamos sequences: As these sequences stem from different studies and techniques (as Reviewer 2 pointed out), it would be interesting to see a sensitivity analysis looking into a subset of the sequences stemming from one study only (i.e., you could take the largest sub-study with available wgs). In my opinion, with this quite biased selection of available sequences, it is not clear which phylogenetic patterns would be expected at all - the current selection of sequences do not belong to one sub-epidemic in a country/region/time-frame/sub-population/etc. Thus the results are also hard to interpret.

Though our focus was on introducing the method rather than exploring specific sub epidemics, we agree with this comment and have performed the analysis as suggested, taking 116 sequences from Pessôa et al. 2016. Our results revealed some differences which we have summarized in the following sections:

Abstract

- Last line of 2^nd^ paragraph: “Under the assumption that clustering from 100\% of long sequences is the most accurate, we obtained 1196 subtype B nFL HIV-1 sequences from the Los Alamos National Laboratory Database and a single-study subset, varied the proportion of long and short sequences in our T-shape alignments, systematically masked all non-pol regions with missing characters in proportional increments, and compared tree similarity and cluster inference among datasets.”
- Last line of 3^rd^ paragraph: “For the subset dataset, we found that the introduction of nFL sequences to the T-shaped alignment improves accuracy in clustering either after a 30% threshold or immediately depending on bootstrap choice.”
- First 2 lines of 4^th^ paragraph: “Our new approach and results suggest that using T-shape alignments to mix HIV-1 sequences of different lengths can improve phylogenetic and clustering accuracy, with needed nFL proportion depending on analysis goals.”

Methods Lines 60-65:

“To address potential biases arising from dataset heterogeneity and sequence source, we additionally created a subset of the filtered nFL genome alignment comprising of the 116 subtype B nFL genomes sequenced in [21] (subset dataset), selected because it is the largest subset of sequences from within a single study and contains geographic linkage information as well. This subset dataset served as a sensitivity analysis.”

Results Lines 137-140:

“For the subset dataset, the wgs40 through wgs99 mixtures show decreasing distance to wgs100 (Fig 3), and the one-tailed paired t-test showed that at wgs40 the distance to wgs is closer than pol to wgs (p-value 0.002534), but mixtures with lower wgs proportions are not.”

Results Lines 168-174:

“For the subset dataset, at bootstrap thresholds of 90, 95, and 99 the mean number of clusters stays around the same until wgs50, then increases as wgs proportion increases from there (Fig 7, S2 Table). At bootstrap thresholds of 70, 80, and 85 the mean number of clusters appears to decrease slightly or stay about the same as wgs proportion increases up to wgs50, then increase slightly. Mean cluster size appears to increase steadily as wgs proportion increases. This provides some indication that increasing wgs sequence proportion after wgs50 can lead to detecting more and larger clusters.”

Changed the subtitle at line 176 to: “Clustering improves after wgs50 for full dataset, and depends on bootstrap threshold for subset dataset”

Results lines 188-192:

“For the subset dataset, at bootstrap thresholds of 70 and 99, AMI increases with the addition of wgs sequences, while at the other bootstrap thresholds, AMI increases after wgs30 based on t-test (Fig~\ref{fig9}). At all other bootstrap thresholds, the AMI increases immediately. We note that the AMI is relatively low across all bootstrap thresholds – this result is expected because of the exponentially large number of possible clusters.”

Discussion lines 246-253

“We found that for large datasets with many unrelated sequences, at lower proportions of nFL genome sequences, the T-shape alignment does not seem to offer any improvement over the use of just pol. For smaller datasets the T-shape alignment can offer improvement over the use of just pol but the proportion of nFL genome sequences depends on choice of bootstrap threshold as well. The difference between the large dataset and smaller dataset could be due to the larger dataset coming from different studies and techniques, while the smaller study comes from a singular region.”

Discussion lines 259-260:

“The nFL proportion threshold depends on both dataset features and on bootstrap threshold.”

Discussion lines 276-284:

“Specifically, in molecular HIV cluster analyses, mixing nFL HIV-1 genome sequences with partial pol sequences may be advantageous only if the proportion of nFL genome sequences exceeds a specific threshold. The threshold appears to differ based on dataset features. In analyses of large datasets with many unrelated sequences, improvement occurs when the majority of sequences are nFL, but in more local settings a lower proportion of nFL sequences may be sufficient. Future work will look into how the T-shaped alignment performs on a Rhode Island regional dataset, as regional clustering may provide greater insight into transmission networks that public health interventions can target [32-35].”

Discussion lines 288-293:

“For smaller datasets with many sequences from the same region, bootstrap threshold affects the proportion of nFL sequences needed to improve accuracy as well. At the most relaxed and strictest bootstrap thresholds (70 and 99), the addition of nFLsequences intoa T-shaped phylogeny always improves accuracy, while at thresholds in-between, at least 30% of nFL sequences may be needed to improve accuracy.”

In addition, we have added Figures 3, 5, 7, 9, and 11, reflecting the addition of the analyses of the subset dataset.

5) Cluster Definition: You defined clusters as matching only if all members were the same. This is a very strict definition, and in my opinion might be too strict. E.g., if you have a cluster with 10 members, even if this cluster is still there with only one member difference, this would lead to a "non-match". I suggest to at least run a sensitivity analysis applying other cluster definition, e.g. more than 50%, or more than 2/3 of the members being the same. The result presented in Figure 4 might be a consequence of this strict definition: It is concerning that already when comparing all wgs to wgs90, the AMI is only between 0.3 to 0.5. If the AMI is anyway quite low, does the difference between the lower wgs values (wgs90, wgs80, wgs70,...) matter so much? It seems that even 10% missing wgs is leading to a much worse result. To understand this better: could you include results of wgs99, wgs95 or other intermediate values as well to understand this "jump" at the beginning? This also questions the whole "take-home message" that at least 50% wgs is beneficial: the actual changes in AMI are minimal.

Thank you for this suggestion. We have added to the manuscript results from using both the overlap coefficient and the results of intermediate values at wgs95 and wgs99. This is reflected in the methods section described in the response to comment (3) above, and also in lines 196-203:

“We additionally measured the overlap coefficient due to its ability to capture subset relationships where one cluster is contained within another. We plotted the proportion of wgs100 clusters recovered at two different overlap coefficient thresholds: 50% and 66% (Fig~\ref{fig10} and Fig~\ref{fig11}). If a cluster in a mixture phylogeny had an overlap coefficient of at least 50% or 66% with a cluster in the wgs100 tree, then we considered the cluster recovered in the mixture phylogeny. We found that for the full dataset (Fig~\ref{fig10}), the pattern of improvement followed as previously, with at least 50% of sequences needing to be nFL before the proportion of recovered wgs100 clusters improved in accuracy relative to an all pol phylogeny. For the subset dataset (Fig~\ref{fig11}), the increase in proportion of recovered wgs100 clusters varied depending on bootstrap or overlap coefficient threshold, but generally increased steadily after 30%, if not before. With this metric the proportion of clusters recovered was generally higher, with recovery proportion approaching 100% at all bootstrap and overlap coefficient thresholds for the subset dataset.”

6) Discussion: please do not use the term "phylodynamic" here, as no dynamic aspect (e.g. cluster growth) was investigated

We have updated the manuscript on line 259 to use the word “phylogenetic” instead of phylodynamic:

“We assessed a T-shape alignment approach that can combine sequences of different lengths into phylogenetic analyses and cluster inference and does not require new software implementations.”

**Reviewers' comments:**

**Comments to the Authors:
Please note here if the review is uploaded as an attachment.**

**Reviewer #1:**

The study by Guang et al assessed the impact of having sequences with varying length on clustering analysis, or more specific how much improvement including full-length sequences can have on the overall clustering outputs. This is an important aspect of clustering, particularly when it is used for public health use in HIV, where information on clusters may be used to inform policies. Thus, accuracy is important. Overall, it is a neat study looking at a common problem in sequence analysis. The study design seems sound, though I am not familiar with the Robinson-Foulds distance and the adjusted mutual index. Thus, I cannot comment on the results for these.

Though, this is a computational journal I struggled to fully understand the study and believe the manuscript could benefit from more detail and clarifications. Also, how does the IQTree algorithm deal with missing data, for example does it ignore positions with incomplete coverage? I may have missed it but some explanations on that would be helpful. Apologies if I missed this, but did including wgs improve bootstrapping support overall?

The IQTree algorithm treats gaps and missing characters as having no information, similarly to most other maximum likelihood-based software such as RAxML and PhyML. Assuming by positions with incomplete coverage you mean sites where some of the taxa do not have information (such as the arms of the T in our T-shaped alignments), IQTree will not ignore those positions, but rather have the same likelihood for that site as if the missing taxa were not there. This is part of the standard maximum likelihood algorithms for phylogeny as described originally by Felsenstein. We have now included some details to try to clarify this since our study is about the effects of missing site data and understandably, knowing how ML phylogenetic methods handle missing data would be helpful.

“They are at lines 79-81: “The IQTree algorithm treats gaps and missing characters as having no information, similar to most other maximum likelihood-based software such as RAxML and PhyML, and thus a site with missing taxa will have the same likelihood as if the missing taxa were not there.”

Minor comments:
Lines 74 to 79 could be re-written to clarify the outcomes. I didn’t understand fully what the distance along y-axis represents and what the distance. I understand how the points move along the axis from wgs20 to wgs90 but not why pol and wgs10 are ‘off’ the line, as in wgs10 is worse than pol?

Thank you for this comment. We have reworded the section with the following revision: “Plotting mixtures onto the two-dimensional Principal Coordinates Analysis (2D PcoA) projection, percent variance explained over both axes (the measure of how much the first 2 principal coordinates contribute to variation in the distance matrix) was fairly low (Fig~\ref{fig4}, 10.98\% x-axis+3.25\% y-axis; Fig~\ref{fig5}, 24.4\% x-axis+3.63\% y-axis). For both the full dataset and the subset dataset, along the first (X) variance dimension, similarity to wgs100 directly increased as proportion of wgs sequences increased, (moving from right to left on the X-axis) suggesting that there is a direct relationship between increasing the proportion of wgs sequences and similarity to the wgs100 tree. Along the second variance dimension (Y-axis), similarity to wgs100 did not directly increase as proportion of wgs sequences increased, with the points forming a u-shape instead. Additionally, for the full dataset trees from different mixtures clearly clustered and separated on the 2D projection. For the subset dataset, trees from different mixtures did not clearly cluster at lower proportions of wgs. This suggests that while increasing the proportion of wgs sequences is the dominant factor and does linearly increase similarity to wgs100, there is a second non-linear factor impacting phylogenetic distance.”

Lines 253 to 260 not very clear

We have attempted to clarify the purpose with the following revision, lines 94-99: “The Robinson-Foulds distances give us an idea of how similar mixture trees and the wgs100 tree are, but not how similar mixture trees are to other mixture trees. To further visualize and assess the similarity between phylogenies inferred from the different mixtures, we computed the Clustering Information Distance from the R package TreeDist as the tree distance metric between all pairs of trees, based on the recommendations outlined in [25], then projected the distance matrix onto 2D with a Principal Coordinates Analysis (PCoA).” We hope this revision clarifies the text.

Line 144. Replace ‘developed’ with ‘assessed’ or similar as per my understanding the study did not ‘develop’ the approach, rather compared outputs.

We have replaced the wording as suggested.

Line 244. While sampling dates are important, I don’t think we can stay bootstrap and genetic distances are ‘arbitrary’. I would rephrase this.

We believe the reviewer may be referring to line 215 in the original manuscript. We have updated the line accordingly to the following: “Third, recent work also suggests that bootstrap and genetic distance thresholds are heuristics and proposes using sampling dates to better estimate emerging clusters.”

Line 245. Unclear what exactly was repeated 1000 times? The clustering? The generation of mixed length datasets?

We have created a methods Figure and revised the methods for clarity; please see our response to the editor above.

Figures

Figure 1. It seems the more ‘wgs’ sequences are present the more variation is in the distance comparison (larger confidence intervals), why is that?

Good question. We would suggest that it is due to the longer sequences filling in gaps and thus leading to more variation at those sites, especially as in HIV-1 the longer sequences include more variable regions such as env.

We have edited accordingly the relevant text at lines at 140-143: “As the proportion of wgs grows, the variation in distances to wgs100 also increases (larger whiskers and boxes), likely due to the longer sequences with more variable regions (e.g. env) filling in gaps and thus leading to more variation at those sites.”

Figure 3 is a bit chaos. Can you separate by bootstrap? Make each panel small, I think that should be sufficient.

Thank you for the suggestion, we have done so and hope the figure is more clear now.

**Reviewer #2:**

Major Points to address:

- Methodological:
- Regarding the entity of the available sequences, the Los Alamos database harbors a variety of methods that have been used to derive those sequences. Unity in sequence acquisition is practically infeasible, however, one major point to address should be that the authors should focus on either sequences derived from viral RNA or proviral DNA and removing the other. Has it been ensured that only viral RNA sequences are used? If not, I recommend doing so, since in cases of mixture, when proviral sequences have been used, an increase in similarity might be spurious due to the increase of similarity of sequences across entity rather than mutual sequence information (e.g. G-A mutations are more common in proviral DNA and might lead to close phylogenetic signal in nFL sequences due to hypermutation instead of genomic evolution). Alternatively, instead of completely removing proviral DNA sequences, the authors could check and include only intact sequences (e.g., using the ProSeqIT tool or HYPERMUT). I acknowledge the fact that this procedure should overall not impact the results substantially, since the analysis is of comparative nature in regards of the initial phylogenetic reconstruction, however, I believe that adapting this would increase the strength of the manuscript.

We thank the reviewer for pointing out this important observation. We had originally not ensured that only viral RNA sequences were used; we went back and assessed the proportion of proviral DNA vs viral RNA in the sequences we downloaded, and found a split of 522 (43.7%) sequences hat were viral RNA, with the rest 674 (56.3%) proviral DNA. We ran HYPERMUT as suggested on all the sequences and found that only 4 of the sequences, or .06% of the proviral DNA sequences were hypermutated. For this reason we chose not to exclude the sequences. This is noted in the manuscript at lines 58-60: “We downloaded a filtered nFL genome alignment of all HIV-1 subtype B sequences published through the end of 2018 from the Los Alamos National Laboratory HIV database (full dataset) as our initial input [19] (Fig 1A) and assessed hypermutation in the alignment with HYPERMUT [20] . We found that out of the 1196 sequences,4 59 were hypermutated. As this was a minimal number, we kept all sequences.”

- The authors introduced gaps when constructing the T-shaped alignments. Since the presented manuscript provides an experiment approach to the methodology, has it been tried to introduce ambiguous nucleotides (‘N’) instead of gap characters and rerun the analysis to assess differences?

IQTree deals with gaps and completely ambiguous nucletodies as N in the same way, as do most other maximum likelihood phylogenetic inference software (e.g. RAxML, PhyML). We have introduced some details on the algorithm to provide some understanding on how such software handles missing data and hopefully clarify points in our manuscript at lines 80-82: ”The IQTree algorithm treats gaps and missing characters as having no information, similar to most other maximum likelihood-based software such as RAxML and PhyML, and thus a site with missing taxa will have the same likelihood as if the missing taxa were not there.”. Please also see comments to Reviewer #1 above.

- When incrementally increasing the proportion of wgs sequences into the T-shaped alignment I did not understand from the method section if only the proportion of the wgs sequences was increased or, starting from the lowest fraction of wgs, partial/pol sequences were ‘filled-up’ incrementally, ensuring that the same wgs are also used in the next step. This would increase comparability and potentially explain the increase in accuracy metric when using 50%.

Thank you for your comment, it appears our methods were rather unclear, we have now clarified our methods with Figure 1 and revisions to the text. Please also see our earlier response to the editor for edits we have made to increase methods clarity in the manuscript.

- Results:
- L115, Besides the wgs50 tree, there is no supporting material for the claims made in this paragraph, which would be necessary for readers to verify the conclusions from these claims. Referring to the other point, one should also distinguish between proviral and viral sequences and check this again.

We thank the reviewer for this comment – upon additional analysis, we discovered that there was indeed a lack of support for our claim, and in fact discovered something somewhat interesting, that there is a slight bias towards pol sequences clustering together. We have included those results in the manuscript accordingly at lines 217-222: “We found a slight bias towards pol sequences grouping together. We calculated the mean proportion of pol sequences in clusters at each mixture proportion, with the expectation that the proportion of pol sequences in clusters should be equal to the proportion of pol sequences in the phylogeny, but found that for every mixture proportion it was higher. For example, for wgs50, the mean proportion of pol sequences in clusters was around 0.64, suggesting a bias towards pol sequences in clusters.”

- Figure 6 is missing.

Our apologies, we have included Figure 6 (now Figure 12).

- Please ensure, also in the supplementary figures, that the x and y axis (and headers) have meaningful labels (e.g. Change ‘Value’ with the metric).

Thank you for noting this, we have provided meaningful labels for the x and y axis now.
