# Supplementary material for: T-shaped alignments integrating HIV-1 near full-length genome and partial pol sequences can improve phylogenetic inference of transmission clusters
Source: PLoS Comput Biol. 2025 Nov 25;21(11):e1013676. doi: 10.1371/journal.pcbi.1013676 (PMC12685204; doi:10.1371/journal.pcbi.1013676)
Supplement: Attachment — Submitted filename: response_to_reviewers.docx [file pcbi.1013676.s007.docx]

**Journal Requirements:**

1) Thank you for stating "Publicly available data at https://www.hiv.lanl.gov/content/sequence/HIV/mainpage.html was used for the analysis." Please confirm whether all data needed to replicate the study's findings are at this link? If so, please update your Data Availability statement to "All data required to replicate the study's findings can be found here  <https://www.hiv.lanl.gov/content/sequence/HIV/mainpage.html>

- This has been changed and updated.

Note: If the reviewer comments include a recommendation to cite specific previously published works, please review and evaluate these publications to determine whether they are relevant and should be cited. There is no requirement to cite these works unless the editor has indicated otherwise.


**Reviewers' comments:**

Reviewer's Responses to Questions

Reviewer #2: This manuscript has improved significantly through all additional analyses and points discussed. Although the study is very thoroughly conducted and has gained improvements, the setup reflects a highly experimental scenario with many uncertainties. Thus, I would suggest to position the attitude towards public health implementations and recommendations for incorporating such nFL genomes even more neutral (e.g. L278-287).

We are pleased that the reviewer is satisfied with our revisions. Thank you for this comment. We have updated the manuscript in multiple places to position recommendations as even more neutral:

- In the Author Summary, we have changed: "Our work underscores the importance of sequence length proportions in genetic analysis, providing valuable insights for public health initiatives.” to: “Our work underscores the importance of sequence length proportions in genetic analysis, providing preliminary insights that may be relevant for future public health initiatives.”
- In the Discussion L278, we have changed: “Our findings have important practical implications for HIV-1 phylogenetic and clustering studies.” To “Our findings have potential implications for HIV-1 phylogenetic and clustering studies.”
- In the Discussion L284-287, we have changed: “Future work will look into how the T-shaped alignment performs on a Rhode Island regional dataset, as regional clustering may provide greater insight into transmission networks that public health interventions can target.” To “Future work will look into how the T-shaped alignment performs on a Rhode Island regional dataset, as regional clustering in a real setting may provide greater insight into transmission networks that could be relevant for informing public health interventions”

Minor points to address:
- L 106/107: You have two unfinished sentences regarding the overlap coefficient.

- Thank you for noting this, we have addressed this and amended to the following sentences at L 106+107: “AMI was chosen due to the unbalanced cluster sizes \cite{Romano2016}, while overlap cofficient was chosen due to its ability to capture subset relationships as it directly measures the proportion of shared elements relative to the smaller of the two clusters.”
- “For the overlap coefficient, a coefficient closer to 1 meant that more members of one cluster are also all members of the other cluster, while a coefficient closer to 0 meant that almost no members of either cluster are members of the other.”
